# Validation of the Iranian Version of the University of California at Los Angeles Posttraumatic Stress Disorder Index for DSM-IV-R

**DOI:** 10.5812/traumamon.10365

**Published:** 2013-10-14

**Authors:** Sholeh Namazi, Maznah Bt. Baba, Halimatun Halaliah Mokhtar, Mohd Sahandri Ghani Hamzah

**Affiliations:** 1 Research Center of Behavioral and Neurological Science, Hormozgan University of Medical Sciences, Bandar Abbas, IR Iran; 2 Department of Counseling Psychology, University of Putra Malaysia, Serdang, Malaysia

**Keywords:** Stress Disorders, Post-Traumatic, Mass Screening, Children

## Abstract

**Background:**

Natural disasters, both expected and unexpected, usually cause widespread injuries and destruction with a large number of survivors, including children. Several studies have shown that children may develop posttraumatic stress disorder after exposure to disasters such as an earthquake.

**Objectives:**

This study aimed to evaluate the screening abilities of the University of California at Los Angeles Posttraumatic Stress Disorder Index for DSM-IV (Revision 1) (UCLA PTSD for DSM-IV) among Iranian school-aged children.

**Patients and Methods:**

Twenty months after the Qeshm Island 6.1-magnitude earthquake in 2008, we screened 50 students aged between 7 and 12 years for posttraumatic stress disorder using the UCLA PTSD INDEX for DSM-IV. A Structured Clinical Interview for DSM-IV criteria was used as the gold standard.

**Results:**

The internal consistency for all the scales was good and Cronbach’s coefficient for the overall items was 0.76. The sensitivity of this questionnaire was high (0.96), while its specificity was moderate (0.50).

**Conclusions:**

The study findings showed that the Iranian version of UCLA PTSD Index for DS-MIV-R was appropriate for screening PTSD in children.

## 1. Background 

In the APA Dictionary of Psychology (1), post traumatic stress disorder (PTSD) is described as a disorder which occurs when an individual lives through or witnesses an event in which one believes that there is a threat to his/her life, physical integrity and safety or experiences fear, terror, or helplessness. According to the DSM-IV-TR (2), PTSD has been recognized as a potential consequence of traumatic event exposure in which, there is an experience of fear, horror, or hopelessness. The essential criteria for clinical diagnosis of PTSD include persistent re-experiencing, persistent avoidance/numbing, and persistent symptoms of increased arousal. Since 1987 when the diagnosis of PTSD was extended to children and adolescents in DSM-III, different methods have been developed for identifying children's reaction to traumatic events (2). In view of children's cognitive development, they may have different interpretations of trauma and consequently, a variety of expressions and presentations of PTSD (3). Compared to preschoolers, school-aged children exhibit more PTSD symptoms and have a greater understanding of traumatic experiences (4). This is due to their higher level of cognitive development compared to preschoolers. However, the symptoms continue to be present in behaviors, such as regressions; e.g. bed wetting, clinging behavior or anxious attachment, and refusing to go to school (5), decreased regulation of emotions, and increase in expressing externalizing or internalizing behaviors, such as fighting with other children, withdrawal from others, decreased attention, and poor school performance (5, 6). In addition, school-aged children may not be able to interpret somatic, affective experiences of PTSD in an abstract manner, such as anxiety and re-experiencing and as a result, describe these experiences by reporting concrete physiological complaints, including abdominal pain and headaches (6). Re-experiencing is often presented as a detailed enactment of the traumatic event or a preservative, verbal description with a lack of appropriate affective expression (7). Fear of going to sleep or being alone, sleep disturbance, clinging to others, and event-specific fears have been reported, as well(5, 8). Traumatic play, at this age, is more sophisticated and includes specific themes, often writing, drawing, and pretending and becomes script-governed (7, 9). Studies of trauma sequelae have rarely described cognitive temporal distortions (8). Reduction of interest in and less enjoyment of normal activities, greater feeling of estrangement from others, and staying inside to be near protective adults is common among the children exposed to trauma (10). Terr noted that children show a high prevalence of "omen formation”, i.e. they believe that specific "signs" warn about the reoccurrence of the traumatic event and that if they are on watch, they will be able to recognize the "omens" and predict the future disasters (9). Since identifying PTSD in children by DSM-III (1987), several measures were designed for assessing PTSD symptoms in children. Of course, most of these measures were the adapted versions of adult PTSD instruments with simplified language and concepts (11). The UCLA PTSD Index for DSM-IV developed by Rodriguez, Steinberg, and Pynoos (12) is a revision of the UCLA PTSD Reaction Index (UCLA PTSD-RI) developed by Pynoos, Rodriguez, Steinberg, Stuber, and Frederick (13). It consists of a child and parent version. This instrument assesses the childrens' and adolescents' reactions to trauma. It can be used as a self-report questionnaire (completed on paper, suitable for one to one or group administration), or be given verbally where questions are read to the child. In addition, the test has excellent psychometric properties and has been used across a variety of trauma types, age ranges, settings, and cultures (14). The internal consistency of the questionnaire was confirmed by Cronbach's coefficient of 0.90 and the test re-test reliability over different versions has ranged from good to excellent. A study reported a test-retest reliability coefficient of 0.84 for the DSM-IV version (15). Besides, its convergent validity was 0.82 in comparison with the Child and Adolescent Version of the Clinician-administered PTSD Scale. Furthermore, a cut-off point of 38 had a sensitivity of 0.93 and specificity of 0.87 in detecting PTSD (16, 17).These self-report instruments can be administered as brief self-report screening tools to identify both exposure to traumatic events and all DSM-IV PTSD symptoms including acute stress disorder (ASD) and post-traumatic stress disorder in school-aged children and adolescents who report traumatic experiences. The items of the UCLA PTSD Index for DSM-IV are matched to the DSM-IV criteria and can provide initial PTSD diagnostic information. However, these instruments were not intended to replace a structured clinical interview to definitely establish a PTSD diagnosis. Instead, the instruments were meant to be used to quickly and efficiently screen PTSD symptoms in children and adolescents who have experienced a traumatic event and provide information regarding the frequency and severity of these symptoms. The content of the questions imply clinical and research experience regarding how to evaluate exposure to traumatic experiences for both children and youth, how they express their subjective reactions during these experiences and how the traumatized children and youth describe their experiences of the PTSD symptoms. A posttraumatic severity score is computed as the sum of the responses to 20 items. Also, three separate scores can be computed for these 22 items for intrusive symptoms, avoidance/numbing symptoms, and arousal symptoms. All three versions of the UCLA PTSD Index for DSM-IV are organized in the same format. The child version contains a total of 20 questions. On the contrary to the first 19 questions, question 20 assesses a common problem reported by traumatized children, namely a fear that the traumatic event will recur. The parent version contains a total of 21 questions, which are identical in content to the child version with one exception. Question 21 has been added to the adult version in order to assess the DSM-IV PTSD symptom of repetitive traumatic play, an alternate expression of criterion B1 in children. A parallel item was not included in the pediatric version since the traumatic etiology of repetitive play is thought to occur outside the awareness of the child.

## 2. Objectives

In this study, we standardized the UCLA PTSD Index for DSM-IV-R for an Iranian population. 

## 3. Patients and Methods

### 3.1. Pilot Study to Determine Instrument Reliability

The original language of the instrument was English; therefore, the instrument had to be translated into Persian. The principal investigator initially translated the UCLA PTSD Index for DSM-IV (child and parent versions) into Persian. Then, the translation was reviewed by a Persian-speaking individual with a M.A. degree in English and back translated into English by an Iranian-American individual. Finally, an Iranian-American geriatric psychiatry fellow at Yale University, USA reviewed the material and confirmed the similarity of the Persian and the English forms in terms of semantic and linguistic aspects. After face validity was approved by five experts, the reliability of this instrument was tested through a pilot study. The pilot study was conducted four weeks after the 4.9 Richter earthquakes in Bandar Abbas, Iran in December 2009. The main purpose of the pilot study was to assess the internal validity of the Persian form of the UCLA PTSD Index for DSM-IV. An elementary school was selected via cluster sampling and 30 students in this school were randomly selected from different grades. It should be mentioned that the researcher had been trained in administrating the UCLA PTSD Index for DSM-IV through a video training presentation on the following site and trained the research evaluator based on the Index manual (18). The UCLA PTSD Index for DSM-IV questionnaire was given to the students and Cronbach’s coefficient of 0.86 was obtained for the overall items. 

### 3.2. Participants and Procedure

After the pilot study, another research was conducted in two elementary schools in the villages of Zeinabieh and Gorbadan, 35 km west of Qeshm Island, south of Iran. This island was hit by a 6.1-magnitude quake on September 10^th^, 2008. There are only two elementary schools in these two villages and both are co-educational consisting of both male and female students between 7 to 12 years-old. Twenty months after the earthquake, initial screening of the participants began by short interviews from all the 311 students in both schools regarding their reaction to the earthquake and their current symptoms based on the PTSD Pediatric Emotion Disorder checklist (PEDS) (19). Overall, 50 children were selected in the initial screening and their parents were briefly interviewed with regards to the possibility of the occurrence of PTSD symptoms. Thereafter, with their parents’ consent, the children were asked to respond to the UCLA PTSD Index for DSM-IV (Child version). Overall, 38 participants had PTSD based on UCLA PTSD Index for DSM-IV. A diagnostic interview based on the DSM-IV-TR structured interview was conducted by a psychiatrist to identify the children who met the initial criteria and 28 children were diagnosed as PTSD based on the clinical interview. 

## 4. Results 

Cronbach’s coefficient was calculated for the sample of 50 elementary students and the results showed a Cronbach’s coefficient of 0.76, 0.80, 0.72, and 0.61 for the overall items, criterion B, criterion C, and criterion D, respectively, which are all acceptable values. Properties of Iranian version of UCLA PTSD for DSM-IV-R are presented in Table 1. Data regarding test results and true status of the subjects being tested through clinical interviews are displayed in Table 2. 

**Table 1.** Properties of Iranian Version of UCLA PTSD for DSM- IV-R Standardization

	Parameters
**Standardization sample**	fifty children exposed to earthquake
**Age, y**	7 - 12
**Face validity**	checked by five experts
**Validity**	0.71 agreement with DSM-IV diagnosis
**Reliability (Cronbach’s α)**	0.07 overall items
	0.80 criterion B
	0.72 criterion C
	0.61 criterion D
**Sensitivity**	0.96
**Specificity**	0.50

**Table 2.** Test Results and True Status of Subjects

PTSD Determined by UCLA PTSD for DSM-IV	PTSD ^a^ Determined by Gold Standard, Clinical Interview, No. (%)		
	Positive	Negative	Total
**Positive**	27 (54)	11 (22)	38 (76)
**Negative**	1 (2)	11 (22)	12 (24)
**Total**	28 (56)	22 (44)	50 (100)

^a^Abbreviations: PTSD, post traumatic stress disorder

## 5. Discussion

The current study aimed to evaluate the validity and reliability of UCLA PTSD Index for DSM-IV questionnaire in a sample of Iranian PTSD children. It is the first psychometric study for this questionnaire in Iran. Based on the results, the sensitivity of the questionnaire was high (27/28 = 0.96) however its specificity was moderate (11/22 = 0.50). The false negative rate was 1/28 = 0.04 and the false positive was No. = 11/22 = 0.50. The high sensitivity and moderate specificity of the questionnaire indicates its good quality for screening. UCLA PTSD Index for DSMIV-R is specifically useful for public health when a large population is exposed to a traumatic event, like a natural disaster or war, in which a high prevalence of PTSD is predictable and probably the majority of children with PTSD need to be correctly identified in order to be referred for an in-depth assessment. The results of this study were in line with the previous research implemented by showing high levels of sensitivity and specificity for UCLA PTSD Index for DSM-IV (15-17). The small number of participants requires cautious interpretation of the results of the study. The main reason for the small sample size was due to the small population of children who had clinical features of PTSD about 20 months after the earthquake experience. In this study, all the children experienced the same single traumatic event, namely an earthquake. Therefore useful information might be missed if a child is exposed to multiple or chronic/repeated trauma, like child abuse. Future research is necessary to be conducted among victims of other types of traumatic experiences. Although face and content validity were generally approved by five experts, lack of quantitative content validity is another limitation of this study. In conclusion, UCLA PTSD Index for DSM-IV is an instrument with good psychometric qualities in identifying PTSD and its different criteria among children can be used for all PTSD symptoms. 

## References

[1] Vanden Bos GR, American Psychological Association (2007). A.P.A. dictionary of psychology..

[2] American Psychiatric Association (2000). Diagnostic and Statistical Manual of Mental Disorders..

[3] Scheeringa MS, Zeanah CH, Myers L, Putnam FW (2005). Predictive validity in a prospective follow-up of PTSD in preschool children.. J Am Acad Child Adolesc Psychiatry..

[4] Aysun D (2004). An examination of adolescents' post-disaster experience and reaction following the 1999 Marmara earthquake..

[5] Yule W (2001). Post traumatic stress disorder in children and adolescents.. Int Rev Psychiatr..

[6] Cook Cottrone C (2004). Childhood posttraumatic stress disorder: Diagnosis, treatment, and school reintegration.. School Psychol Rev..

[7] Johnson MK (2006). Posttraumatic stress in children and adolescents following the 1994 Northridge, CA earthquake..

[8] Cohen JA, Berliner L, Mannarino AP (2000). Treating traumatized children: A research review and synthesis.. Trauma Violence Abuse..

[9] Terr LC (1991). Childhood traumas: an outline and overview.. Am J Psychiatry..

[10] Cohen JA, Deblinger E, Mannarino AP, Steer RA (2004). A multisite, randomized controlled trial for children with sexual abuse-related PTSD symptoms.. J Am Acad Child Adolesc Psychiatry..

[11] Coffman S (1998). Children's reactions to disaster.. J Pediatr Nurs..

[12] Hawkins SS, Radcliffe J (2006). Current measures of PTSD for children and adolescents.. J Pediatr Psychol..

[13] Roussos A, Goenjian AK, Steinberg AM, Sotiropoulou C, Kakaki M, Kabakos C (2005). Posttraumatic stress and depressive reactions among children and adolescents after the 1999 earthquake in Ano Liosia, Greece.. Am J Psychiatry..

[14] Steinberg AM, Brymer MJ, Kim S, Briggs EC, Ippen CG, Ostrowski SA (2013). Psychometric properties of the UCLA PTSD reaction index: part I.. J Trauma Stress..

[15] Rodriguez N, Steinberg AS, Saltzman WS, Pynoos RS (2001a). PTSD Index: psychometric analyses of the adolescent version.. Symposium conducted at the Annual Meeting of the International Society for Traumatic Stress tudies.

[16] Pynoos RS, Rodriguez N, Steinberg AS, Frederick C (1998). The UCLA PTSD Reaction Index for DSM IV (Revision 1)..

[17] Rodriguez N, Steinberg AS, Saltzman WS, Pynoos RS (2001b). PTSD Index: preliminary psychometric analyses of child and parent versions.. Symposium conducted at the Annual Meeting of the International Society for Traumatic Stress Studies..

[18] Saylor CF, Swenson CC, Reynolds SS, Taylor M (1999). The pediatric emotional distress scale: a brief screening measure for young children exposed to traumatic events.. J Clin Child Psychol..

[19] Posttraumatic Stress Disorder Reaction Index (UCLA-PTSD RI)..

